# Effects of strategies to improve general practitioner-nurse collaboration and communication in regard to hospital admissions of nursing home residents (*interprof* ACT): study protocol for a cluster randomised controlled trial

**DOI:** 10.1186/s13063-020-04736-x

**Published:** 2020-11-05

**Authors:** Christiane Müller, Berit Hesjedal-Streller, Nina Fleischmann, Britta Tetzlaff, Tina Mallon, Martin Scherer, Sascha Köpke, Katrin Balzer, Linda Gärtner, Indre Maurer, Tim Friede, Hans-Helmut König, Eva Hummers

**Affiliations:** 1 Department of General Practice, University Medical Center Göttingen, Humboldtallee 38, D-37073 Göttingen, Germany; 2 Nursing Science, Fulda University of Applied Sciences, Building 31, Room 122, Leipziger Straße 123, D-36037 Fulda, Germany; 3 Department of General Practice and Primary Care, University Medical Center Hamburg-Eppendorf, Martinistraße 52, D-20246 Hamburg, Germany; 4 Institute of Nursing Science, University Clinic Cologne, Gleueler Straße 176-178, D-50935 Köln, Germany; 5 Institute for Social Medicine and Epidemiology, Nursing Research Group, University of Lübeck, Ratzeburger Allee 160, Haus 50, D-23538 Lübeck, Germany; 6 Chair of Organization and Corporate Development, Georg-August-University Göttingen, Platz der Göttinger Sieben 3, D-37073 Göttingen, Germany; 7 Department of Medical Statistics, University Medical Center Göttingen, Humboldtallee 32, D-37073 Göttingen, Germany; 8 Department of Health Economics and Health Services Research, University Medical Center Hamburg-Eppendorf, Martinistraße 52, D-20246 Hamburg, Germany

**Keywords:** Nursing homes, Interprofessional relations, Physician-nurse

## Abstract

**Background:**

In Germany, up to 50% of nursing home residents are admitted to a hospital at least once a year. It is often unclear whether this is beneficial or even harmful. Successful interprofessional collaboration and communication involving general practitioners (GPs) and nurses may improve medical care of nursing home residents. In the previous *interprof* study, the six-component intervention package *interprof* ACT was developed to facilitate collaboration of GPs and nurses in nursing homes. The aim of this study is to evaluate the effectiveness of the *interprof* ACT intervention.

**Methods:**

This multicentre, cluster randomised controlled trial compares nursing homes receiving the *interprof* ACT intervention package for a duration of 12 months (e.g. comprising appointment of mutual contact persons, shared goal setting, standardised GPs’ home visits) with a control group (care as usual). A total of 34 nursing homes are randomised, and overall 680 residents recruited. The intervention package is presented in a kick-off meeting to GPs, nurses, residents/relatives or their representatives. Nursing home nurses act as change agents to support local adaption and implementation of the intervention measures. Primary outcome is the cumulative incidence of hospitalisation within 12 months. Secondary outcomes include admissions to hospital, days admitted to hospital, use of other medical services, prevalence of potentially inappropriate medication and quality of life. Additionally, health economic and a mixed methods process evaluation will be performed.

**Discussion:**

This study investigates a complex intervention tailored to local needs of nursing homes. Outcomes reflect the healthcare and health of nursing home residents, as well as the feasibility of the intervention package and its impact on interprofessional communication and collaboration. Because of its systematic development and its flexible nature, *interprof* ACT is expected to be viable for large-scale implementation in routine care services regardless of local organisational conditions and resources available for medical care for nursing home residents on a regular basis. Recommendations will be made for an improved organisation of primary care for nursing home residents. In addition, the results may provide important knowledge and data for the development and evaluation of further strategies to improve outpatient care for elderly care-receivers.

**Trial registration:**

ClinicalTrials.gov NCT03426475. Initially registered on 7 February 2018.

## Background

Hospital admissions can be straining events for nursing home residents (NHRs) with unclear benefits for their further health progress; they often result in an increased risk of invasive procedures, new decubital ulcers, development of a delirium or potentially invasive interventions [1]. For NHRs with dementia, hospitalisation goes along with a higher risk for physical and cognitive decline, nosocomial infections, falls, increased length of stay in hospital and mortality [2].

In Germany, up to 50% of NHRs are admitted to hospital at least once a year [3–5]. The incidence is comparable to international studies with hospitalisation rates ranging from 9 to 59% [6]. Latest numbers from Germany indicate that around 21 out of 100 NHRs are hospitalised during a quarter at least one time [7], while others report an overall hospitalisation rate of NHRs of 1.4 per person-year [8].

Of NHRs’ transfers to hospitals, around 40% are considered avoidable [9] of transfers to emergency departments even up to 55% [10]. In Germany, 32 of 100 NHRs were hospitalised for ambulatory-sensitive reasons during a year, which means, that these hospitalisations are considered to be avoidable by timely prevention or treatment by outpatient health services ([11]. A recent German study reports 29.6% potentially avoidable hospitalisations of NHRs [12].

In Germany, currently around more than 8,000,000 people are cared for in a nursing home [13]. The medical care of NHRs does not differ from other care-dependent persons with the right of free choice of doctors. The medical care for NHRs is mainly the responsibility of general practitioners (GPs). Within 3 months, over 90% of NHRs receive one or more medical home visits from their GPs [7].

Since January 2019, during the running study, nursing homes have been required by law to make cooperation agreements with physicians in ambulatory care to improve the flow of information between these facilities and GPs or outpatient medical specialists as well as the timely access to physicians when NHRs suffer acute deteriorations of their chronic conditions or acute complications such as fall-related injuries or pneumonia [14].

The quality of nurse-physician collaboration in the medical care for NHRs is assumed to be an important precondition for the prevention of potentially avoidable hospital admissions in this vulnerable population. During the last years, a few qualitative studies on interprofessional collaboration have been carried out in Germany. They show a potential to improve collaboration of nurses and GPs. Nurses assess a smaller number of GPs assigned to individual nursing homes, and GPs’ visits at fixed times as being potentially beneficial for the collaboration between GPs and the health care facility [15]. Nursing staff wishes also for better accessibility of GPs, and improvements in the communication with GPs, both in terms of quantitative contact time and the quality of communication [16]. Altogether, they want to be more respected and “be involved” in medical decision-making [17]. From the GPs’ viewpoint, effective and sustained modes of exchanging information are important, but altogether they report less needs for improvements [16]. With regard to nursing home visits, they strive for a “productive performance”, meaning a subjectively well balanced trade-off between personal efforts and reward [18]. In general, the implementation of viable concepts for quality improvements in the medical care for NHRs highly depends on medical doctors’ willingness to cooperate [19].

To date, research evidence on the effects of interprofessional practice-based interventions on the quality of health services, especially patient-important outcomes, is limited [20]. Previously investigated strategies on preventative measures of hospitalisation of NHRs did not yield consistent results and did not primarily address the interprofessional collaboration between GPs and nursing staff [21]. However, interprofessional interventions showed a positive impact on NHRs’ health (e.g. reduction of depressive symptoms or agitated behaviour in NHRs suffering from dementia). One key to success was the inclusion of resident GPs [22].

A few interprofessional programmes or studies implying the improvement of the interprofessional collaboration among nurses and mainly physicians in the nursing home to reduce hospitalisations have been implemented during the last decade. The US quality improvement programme INTERACT has a broad multi-strategy approach containing, among others, constant analyses of causes of hospitalisation, early detection of change of the NHR’s health and an improvement of communication and documentation [23]. Although first studies were successful in reducing hospitalisation of NHRs [24], the latest large trial showed no reduction of hospitalisations or emergency department visit rates [25]. In a smaller non-randomised Austrian study, a significant reduction of hospital transfers was found after additional qualification of nursing staff and interventions to improve interprofessional communication/collaboration [26]. In recent time, due to the request for proposals of the innovation committee, several interprofessional interventional studies are ongoing in the long-term care setting in Germany, among them are HIOPP-3-iTBX study [27], CoCare [28] and SaarPHIR [29] and the study *interprof *ACT which is presented in this protocol.

In our previous *interprof* study, we developed six measures, the “*interprof* ACT” intervention package, for better collaboration and communication between nursing home staff, especially nurses, and GPs. These measures were identified and created in a qualitative multistep bottom-up process reflecting the perspectives of the involved person groups (GPs, nurses, NHRs and relatives), followed by an exploratory pilot study involving four nursing homes [30]. In this pilot study, the participating facilities could choose measures according to their needs and implemented them for a 3-month period. GPs and nurses evaluated the measures positively with regard to feasibility and acceptance [31]. In the present study, we aim to evaluate the effect of these interventions in a larger group of NHRs.

## Trial objectives

The major aim of this cluster randomised controlled trial is to examine the clinical effectiveness of *interprof * ACT in German nursing homes.

Our *main hypothesis* is that implementation of *interprof * ACT reduces the incidence proportion of hospitalisations of NHRs within 12 months from 50 to 35% (i.e. an absolute reduction of 15 percentage points) compared to the control group, which receives care as usual.

Our *secondary hypotheses* are that the number of NHRs admitted to hospital within a year as well as the number of days in hospital decrease, while NHRs’ quality of life is positively affected. In addition, we will examine the impact of *interprof * ACT on safety outcomes (falls and decubitus ulcers, chronic wounds and pneumonia) and will perform a health economic evaluation. Also, this trial includes a comprehensive process evaluation to explore the intervention’s impact on important intermediate outcomes like interprofessional collaboration and residents’ satisfaction with medical care and to identify preferred implementation conditions in routine care. The process evaluation will be described in a separate study protocol, which is currently under submission.

## Methods/design

### Trial design

**Fig. 1:**
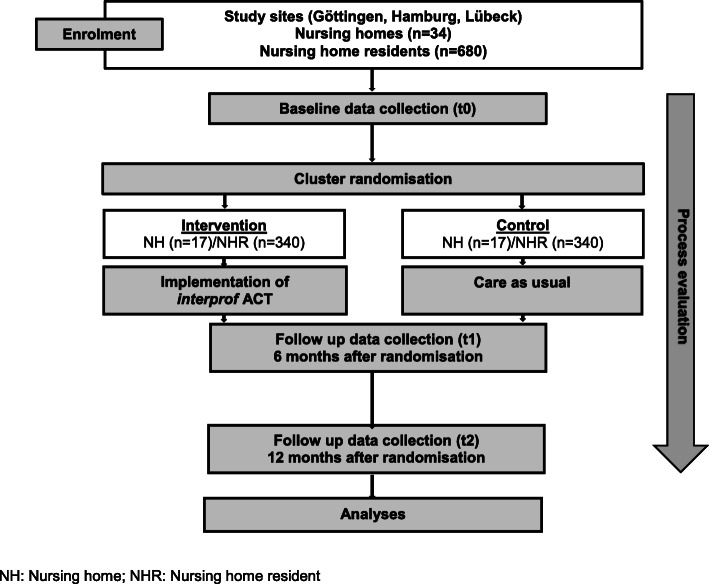
Flow chart providing an overview of important trial steps

The study is a multicentre, cluster randomised controlled interventional study (cluster-RCT). The study centres are Göttingen, Hamburg and Lübeck in Germany. Nursing homes will be randomised pairwise 1:1 to intervention and control group, and follow-up will take 12 months after randomisation. For nursing homes allocated to the intervention group, the *interprof * ACT intervention package will be introduced for a period of 12 months. In nursing homes allocated to the control group, the care for the residents is carried out as usual (Fig. 1).

### Participants and recruitment

#### Inclusion and exclusion criteria

In total, we include 34 nursing homes in the cities and catchment areas of Hamburg (*n* = 16), Lübeck (*n* = 10) and Göttingen (*n* = 8). We will aim to recruit 20 NHRs in each nursing home to form a cluster. A total of 680 NHRs will be enrolled in this trial.

All nursing care institutions providing inpatient care for the elderly according to § 71 SGB XI (social code book XI) are eligible for participation in the study. Inclusion criteria for nursing homes are as follows: a minimum size of 40 NHRs and written consent provided by the nursing home manager prior to randomisation. Nursing homes will be excluded if they currently take part in other projects on interprofessional collaboration.

All NHRs within a cluster are eligible for participation in the study. Inclusion and exclusion criteria can be found in Table 1.

**Table 1:** Inclusion and exclusion criteria for nursing home residents

Inclusion criteria	Exclusion criteria
• At least one GP contact in recent 3 months	• Admission for short term care only
** or**	
Two GP contacts in recent 6 months	
** or**	
Admission to the nursing home during the precedent 6 months independently of documented GP contacts	
• At least 18 years of age	
• Written informed consent by the resident or her/his legal guardian	

#### Recruitment of nursing homes and study participants

Nursing homes will be selected by postal codes from a local facility register. At first, facility managers and nursing directors will be informed in written and in the next step in oral form about the study and receive an invitation to participate. In case of interest, a research team member introduces the study in a personal meeting (Table 2).

**Table 2:**
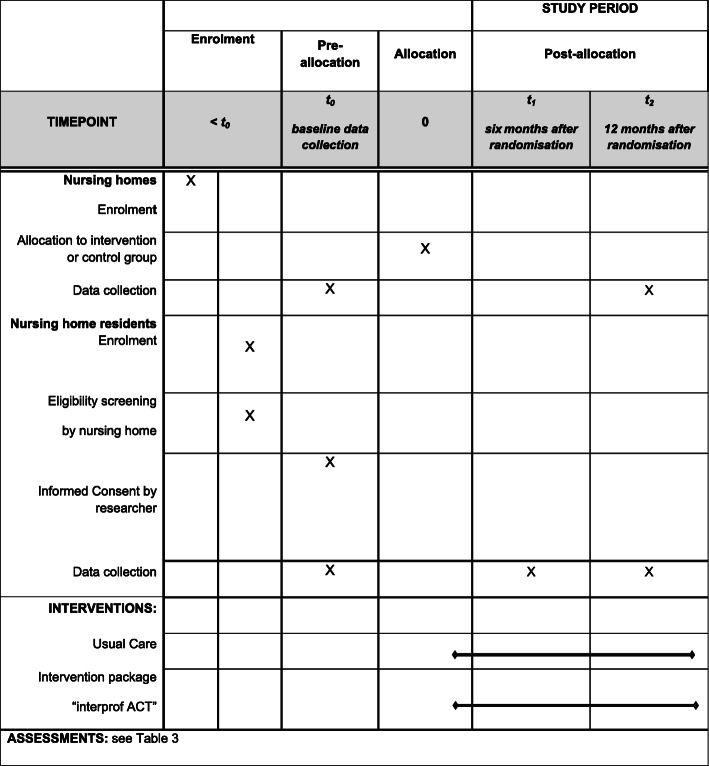
Schedule of enrolment and interventions

Up to 30 residents will be included in order of their consent in each nursing home. A nursing home employee appointed by the nursing home direction will screen NHRs for eligibility. All eligible NHRs or their proxies or legal guardians will receive written information about the aims and the purpose of the study as well as all the information required according to good clinical practice (GCP) rules. This includes written information about the updated version of the General Data Protection Regulation (GDPR). If a NHR/legal representative is willing to participate, a study team member will inform this person directly. Afterwards, the informed consent will be signed by a study team member and the NHR/legal representative. The NHR/legal representative allows the study team to contact the respective GP. NHR’s inclusion will be independent from GP’s decisions on participation. NHRs will be recruited before randomised allocation of the nursing homes to one of the two study groups in order to avoid recruitment bias.

All GPs in the area will initially receive general information about the project, e.g. via existing local GP newsletters or presentations in local GP networks. GPs of the recruited NHRs will be contacted in written and oral form and asked for participation before randomisation.

As incentives, GPs and nursing homes receive respectively an expenditure allowance of EUR 150 per included NHR of the intervention group and of EUR 50 per NHR of the control group. NHRs receive a small thank you gift in case of participation.

### Intervention

#### The interprof ACT intervention package

For nursing homes allocated to the intervention group, the *interprof * ACT intervention package will be introduced. The *interprof * ACT intervention package contains the following components:

1. Use of name badges worn by GPs and nurses during the GPs’ visits
2. Appointment of a contact person: Nursing homes appoint one registered nurse for each unit and GPs one member of their practice staff
3. Mandatory availability: for each of the appointed contact persons via phone and fax (use of a *interprof* ACT standardised fax sheet)
4. Standardised procedures for GPs’ home visits: Visits will either be scheduled weekly or be announced 2 days in advance and arranged within a time slot of about 2 h. If relatives should attend, the appointed contact person informs them about the schedule of the visit. The procedure is structured as follows: The appointed contact person of each facility collects and prioritises the concerns of the NHRs beforehand. On arrival, the GP and the appointed contact person discuss these concerns followed by the actual home visit which might be accompanied by the appointed contact person. In case the appointed contact person was not present during the home visit, a final meeting between the GP and the contact person terminates the procedure or the GP documents clear instructions in the resident’s file. If the nurse accompanies the GP, clear decisions on further procedures are fixed in the resident’s file, too.
5. Support in assigning pro re nata medication: forms including details on symptoms or side effects, dosage and maximum daily dose.
6. Meetings for shared goal setting: Therapy goals specific to each NHR will be approved and documented by all involved parties (e.g. GPs, NHRs, nurses and if desired relatives) in regular intervals (quarterly).

The intervention components will be selected and arranged by nurses, GPs, NHR’s representatives and other participants according to the care facility’s needs in a kick-off meeting. To ensure full and sustained implementation of the *interprof * ACT intervention, different strategies will be combined.

#### Intervention strategies

1. Designated *interprof * ACT agents: Each nursing home should designate an *interprof * ACT agent and a substitute. Acting as in-house change agents, *interprof* ACT agents will plan, facilitate and oversee the implementation of the locally selected *interprof * ACT components in the daily care routines. Together with the facility’s nursing director, they and a member of the study team will prepare the in-house kick-off meeting with all involved parties. The *interprof * ACT agent receives two trainings: At the first training (duration 120 min) directly after randomisation, the *interprof* ACT agents will be instructed by a study team member about the measures. Moreover, they will learn how to obtain the opinion of the colleagues about the *interprof* ACT measures and how to organise the kick-off meeting. The second training (duration 120 min) will be conducted after the kick-off meeting in order to discuss the implementation of the adapted measures as well as the maintenance and supervision. Study assistants will supervise regularly the *interprof * ACT agents by telephone, electronic contacts or face-to-face meetings to facilitate the implementation process. For the first 3 months after randomisation, 2–4 telephone/electronic contacts per month and 1–3 face-to-face meetings in total are planned, which should be reduced to 1–2 monthly telephone/electronic contacts and 1 face-to-face meeting every other month in the remaining study period. The exact timepoints and intervals of contacts are at the discretion of the designated *interprof* ACT agents and the supervising study assistant
2. Involvement of NHRs’ GPs: GPs will be informed in written form and orally by the local research team about the allocation of each facility. GPs caring for NHRs of nursing homes assigned to the intervention group will receive the name of the nursing home’s *interprof * ACT agents and an overview of the *interprof * ACT components. They will be invited to the in-house kick-off meeting with the other involved parties.
3. In-house kick-off meeting involving all parties (nursing home director, director of nursing, local *interprof* ACT agents, head nurse and registered nurses of participating units, GPs of included NHRs, representatives of the home advisory board or/and NHRs, representatives of the relatives advisory board or up to two interested relatives as representatives): At this meeting (duration 120 min), the *interprof * ACT components to be implemented are chosen and agreed on. The discussion is facilitated by an *interprof* ACT agent and a member of the local research team. All meeting attendees discuss and build consensus on the aims and exact content of the selected components and define the methods and practicalities of the awaiting implementation process.
4. Involvement of NHRs: All NHRs and/or their legal representatives receive information about the assignment of the unit/facility in written form.
5. Support for implementation and materials for communication: Each facility is provided with materials to support the implementation such as a model for nametags, documentation forms and materials for announcing *interprof* ACT within the facility.

### Control group

In the control group, standards and procedures of medical care organisation and interprofessional collaboration will not be altered. Nursing staff and GPs will work as usual. The control group care facilities, GPs and NHRs receive information about their allocation to the control group. The information for the care facilities and GPs will also stress the importance of interprofessional collaboration regarding the quality of primary care for NHRs.

After data collection at month 12, control group nursing homes will be offered to implement the intervention, too.

### Outcomes

#### Outcomes (effectiveness)

Primary outcome is the incidence proportion of hospitalisation (residents with at least one hospital admission) within 12 months after randomisation. Secondary outcomes include the total number of admissions to hospital, total number of days admitted to hospital and the usage of other medical services within 12 months (Tables 3 and 4). The study uses the FIMA questionnaire (FIMA: Questionnaire for Health-Related Resource Use in an Elderly) [32] to measure utilisation of health care which will be monetarily valued by standard unit costs [33]. The FIMA was developed to measure health care utilisation in German populations, in particular in the elderly, and is the best available instrument for our purposes, measuring the utilisation of health care providers, for example areas of hospital stays, outpatient visits to physicians and non-physicians, use of pharmaceuticals or out of hour care. It was pilot tested [32], but not systematically validated so far. Moreover, we will collect data on the prevalence of potentially inappropriate medication (according to the PRISCUS list: potentially inappropriate medication for older people) [34]. Quality of life will be assessed by the NHRs themselves or a proxy (staff) depending on the resident’s cognitive state. The Quality of Life-Alzheimer’s disease questionnaire nursing home version (QoL-AD NH) [35, 36] and the standardised instrument developed by the EuroQol Group (EQ-5D-5L) [37] will be used as measures of health-related quality of life. The QoL-AD-NH consists of 15 items, with the possible answers “poor” (item score 1), “fair” (item score 2), “good” (item score 3) and “excellent” (item score 4). It is the only available instrument which allows both self and proxy measurement of quality of life specifically in NHRs with cognitive impairments [35]. We used the culturally adapted German versions [35] of the original instruments for self and proxy ratings, which both demonstrated high internal consistency [38]. For the German versions of the QoL-AD-NH as used in our trial, no psychometric data have been published so far but sufficient estimates of construct validity, internal consistency and inter-rater reliability are reported for proxy ratings by means of the non-NH-specific QoL-AD when applied to German NHR samples [36, 39]. The EQ-5D-5L measures health-related quality of life on five ordinal scales and a visual analogue scale (EQ VAS). Responses on the five scales can be aggregated to a single preference-based index of health-related quality of life using scale-specific weights published by Ludwig et al. [40]. The validity of the EQ-5D-5L has been analysed for several diseases relevant in our patient population, for example heart failure [41], stroke [42], asthma [43], diabetes [44] or COPD [45]. In summary, the studies found that the EQ-5D-5L is a valid instrument that can be used to measure health-related quality of life.

**Table 3:** Overview of study outcomes, time points of data collection and data sources (t1 baseline before randomisation, t1 6 months after randomisation, t2 12 months after randomisation)

Outcomes	Time			Data source
	t0	t1	t2	
**Nursing home residents**				
**Primary outcomes**				
Cumulative incidence hospitalisation	x	x	x	Resident file
**Secondary outcomes**				
Hospitalisations	x	x	x	Resident file
Hospital days	x	x	x	Resident file
Inappropriate medication	x	x	x	Resident file
Adverse events	x	x	x	Resident file
Mortality	x	x	x	Resident file
Medical care	x	x	x	Resident file
FIMA	x	x	x	Resident file
Quality of Life (QoL-AD-NH, EQ-5D-5L)	x		x	Self-administered questionnaire (standardised interview with residents) or proxy assessment by nurses
**Intermediate outcome (process evaluation)**				
Satisfaction with medical care	x		x	Self-administered questionnaire
**Nurses in nursing homes (intermediate outcome for process evaluation)**				
Quality of interprofessional collaboration, including determinants and context factors potentially influencing the implementation and effects of *interprof* ACT (quantitative data)	x		x	Self-administered questionnaire
Process of interprofessional collaboration, including determinants and context factors potentially influencing the implementation and effects of *interprof* ACT (qualitative data)	x	x	x	Non-participatory observations of kick-off meetings and interprofessional collaboration, semi-structured interviews
**Nursing home managers (intermediate outcome for process evaluation)**				
Quality interprofessional collaboration, including determinants and context factors potentially influencing the implementation and effects of *interprof* ACT (quantitative data)	x		x	Self-administered questionnaire
**General practitioners (intermediate outcome for process evaluation)**				
Quality of interprofessional collaboration, including determinants and context factors potentially influencing the implementation and effects of *interprof* ACT (quantitative data)	x		x	Self-administered questionnaire
Process of interprofessional collaboration, determinants and context factors potentially influencing the implementation and effects of *interprof* ACT	x	x	x	Non-participatory observations of kick-off meetings and interprofessional collaboration, semi-structured interviews

**Table 4:** Definition of outcomes

Domain	Specific measurement	Specific metric	Method of aggregation	Timepoint		
				t0	t1	t2
**NH resident**						
**Primary outcomes**						
Hospitalisation	Number of hospitalisation	Within 12 months	Incidence proportion	x	x	x
**Secondary outcomes**						
Number of admissions to hospital	Number of admissions	Within 12 months	Mean	x	x	x
Number of hospital days	Number of hospital days	Within 12 months	Mean	x	x	x
Hospital admissions	Reason for admission, initiation by whom, discharge diagnosis	Within 12 months	Proportion of residents with mentioned measurement categories	x	x	x
Usage of other medical services	Contact (forms: fax, telephone call, home visit, reason, initiation) to GP, specialists, therapists, emergency medical services, use of rehabilitation clinics, transports for medical reasons	Within 12 months	Proportion of residents with at least one use of other medical services	x	x	x
Prevalence of inappropriate medication	Usage of PRISCUS criteria	Within 12 months	Proportion of residents with at least one potentially inadequate medication	x	x	x
Adverse events	Pressure ulcers, chronic wounds, pneumonia, falls	Within 12 months	Proportion of residents with at least one adverse event	x	x	x
Mortality	Death	Within 12 months	Proportion of residents who died	x	x	x
Economical evaluation	FIMA Questionnaire	Within 12 months	Incremental cost-effectiveness ratio and cost savings	x	x	x
Quality of Life-Alzheimer’s Disease	QoL-AD-NH	Within 12 months	Mean (score 1–4)	x		x
EuroQol-5 Dimension-5 Level	EQ-5D-5L	Within 12 months	Mean (5-digit number)	x		x
Satisfaction with medical care	Self-administered questionnaire	Within 12 months	Mean	x		x
**Nurses**						
Interprofessional collaboration	Self-administered questionnaire	Within 12 months	Mean^a^	x		x
**NH manager**						
Interprofessional collaboration	Self-administered questionnaire	Within 12 months	Mean^a^	x		x
**General practitioner**						
Interprofessional collaboration	Self-administered questionnaire	Within 12 months	Mean^a^	x		x

^a^Various sub-scores, detailed information on specific measures are reported in the separate protocol of the process evaluation (currently under submission)

Depending on the NHRs’ cognition, either self-reported judgments by the NHRs or proxy judgments by nurses will be retrieved for this quality of life assessment. To appraise residents’ ability to answer questionnaires and as an indication of cognitive impairments, the Dementia Screening Scale (DSS) will be applied as proxy measure by nursing home staff [46, 47]. The Dementia Screening Scale (DSS) comprises seven items in two fields of cognitive functioning: memory (items 1–4) and orientation (items 5–7). The items are assessed using a three-level Likert scale. Per item 0 to 2 points can be awarded. In the literature, two possible cut-off points (2/3 or 3/4) are given; in *interprof* ACT, we chose the cut-off point 3/4. For this threshold, an overall correct classification (OCC) of 87.4%, a sensitivity of 88.8%, a specificity of 86.6% and a positive predictive value (PPV) of 81.6% are reported [46].

Mortality is included as an exploratory outcome. In order to assess any potential negative consequences for the NHR’s health, hospital discharge letters and residents’ nursing records will be analysed for adverse events (falls, decubitus ulcers, chronic wounds, pneumonia).

#### Process evaluation

The trial includes a comprehensive mixed methods process evaluation based on existing frameworks for the evaluation of complex interventions [48, 49]. The objectives of this evaluation are to assess the uptake and implementation of chosen and locally adapted *interprof * ACT measures, to identify relevant barriers and facilitators and to explore the down-stream effects on the quality of interprofessional collaboration and medical care for NHRs which are regarded as important intermediate outcomes. For the latter purpose, various dimensions of nurse-GP (interprofessional) collaboration and communication as well as residents’ satisfaction with medical procedures and GP’s contacts are measured. Data are collected at several time points from all parties involved in the implementation of the intervention under evaluation and the residents, using a combination of standardised questionnaires, minutes of the kick-off meetings and each supervision encounter, non-participating observations and semi-structured interviews (Table 3). A detailed study protocol including an overview of all outcome domains and measures used for the process evaluation is under submission.

#### Outcomes (health economics)

Primary outcome of the health economic evaluation is the incremental cost-effectiveness-ratio, i.e. the ratio of the difference in costs between intervention and control group and the difference in effectiveness between intervention and control group. In the economic evaluation, primary measure of effectiveness will be quality-adjusted life years (QALYs) based on the EQ-5D-5L [37]. Avoided hospital admissions will count as secondary measure of effectiveness. Data on usage of medical services will be rated monetarily with standardised cost unit rates.

### Primary endpoint

Health care utilisation is assessed through the incidence proportion of hospitalisations within 12 months: Any transmission of a NHR to a hospital resulting in a nursing home absence for more than 24 h, or if data on exact times is not available, including a change of date will count as hospitalisation.

### Secondary endpoints

1. Mean number of hospitalisations per resident within 12 months
2. Mean number of hospital days within 12 months
3. Mortality (proportion of residents dying) within 12 months
4. Proportion of residents with at least one potentially inadequate medication at baseline and at follow-ups after 6 and 12 months
5. Proportion of residents with at least one adverse event within 12 months
6. Mean NHRs’ quality of life at baseline and for follow-up at 12 months
7. Proportion of residents with at least one use of other medical services within 12 months
8. Process evaluation: quality of nurse-GP collaboration and communication
9. Economic evaluation: efficiency (incremental cost-effectiveness ratio) and cost savings from payer and societal perspective

### Sample size/power calculation

The proportion of NHRs hospitalised within 12 months is estimated to be up to 50% [3–5]. An absolute reduction of 15 percentage points from 50 to 35% is considered a relevant intervention effect. A sample size of 170 NHRs per group (340 in total) yields a power of 80% at a two-sided significance level of 5%. Adjustment for 20% dropout results in a total sample size of 425 NHRs. If a NHR moves away or dies during the study, the data observed up to this point will be included in the analyses. NHRs that left or died will not be replaced. This is a cluster randomised trial. To account for possible correlations within clusters, the sample size is factored up by the design effect. Assuming an average cluster size of 20 NHRs per home and an intra-cluster correlation (ICC) of 0.021 results in a design effect of 1.4 [50] and a total sample size of 600 patients (30 clusters in total (15 clusters per group) with an average of 20 NHRs per cluster). Clusters (nursing homes) that do not recruit any patients are to be replaced. Anticipating that about 4 clusters will drop out, we will randomise a total of 34 clusters. Originally, we planned to include 18 nursing homes in Hamburg, 8 in Lübeck and 8 in Göttingen. Due to recruiting problems in Hamburg, Lübeck recruited two additional nursing homes, resulting in 16 nursing homes in Hamburg, 10 in Lübeck and 8 in Göttingen. Since there is some uncertainty in the planning with regard to the hospitalisation rates in nursing home ICC and dropout, we conduct a sample size review once the first 300 residents were recruited with a view to adjust the sample size [51].

### Cluster randomisation

After recruitment of NHRs and GPs and baseline data collection (T0), participating care facilities are randomised to the intervention or control group with an allocation ratio of 1:1. The random sequence is generated by the Department of Medical Statistics at UMG (University Medical Center Göttingen); the trial statistician oversees this process. The department is not involved in either the recruitment, the intervention or data collection. Participating care facilities or other members of the trial team will not have access to the randomisation list (randomisation concealment). Random allocation of the care facilities to the intervention groups will proceed in pairs within a research centre, with one care facility being allocated to the intervention and the other to the control group.

The trial statistician (or designee) informs the nursing home management directly via email about the group allocation. The local research team and the lead study office receive this information at the same time.

### Blinding

Due to the nature of the intervention, blinding of the nursing home staff and the research staff will not be possible with the exemption of the primary outcome assessors. Blinding of primary outcome assessment will be guaranteed, as study assistants blinded towards the nursing homes’ group assignment will extract data on incidence of hospitalisation from residents’ files. Also, treatment allocations will be concealed (see above). The standardised extraction procedure to gather data on the usage of medical services from the nursing home resident’s files including documents (e.g. hospital reports) has been proven feasible and reliable [52, 53]. Since the trial statistician oversees the randomisation process, the trial statistician is not blinded to group allocations.

### Data management

All data collected from the nursing home and NHRs in the main trial will be pseudonymised. The personal data of the study participants will be kept separately from the study data. A retrospective correlation to a person is only possible with the help of a “key” which is maintained in the study centre. The data is entered into the electronic database according to the four-eye principle. Pseudonymised data is stored paper-based in the participating study centres and electronically in the database on a server. To ensure data quality, a plausibility check of the data is carried out by an independent monitor. Study data in the process evaluation will be recorded both pseudonymised and anonymous. All original data will be stored for 10 years and destroyed afterwards. In principal, data will be handled according to current data protection law.

### Statistical analyses

The primary analyses follow the intention to treat (ITT) principle, i.e. all randomised nursing homes will be analysed in the group they were randomised to. In case dropout nursing homes discontinue the study, all available data will be used in the analyses. The primary endpoint incidence proportion of hospitalisations within 12 months will be analysed using a generalised linear mixed effects model (GLMM) with fixed effects for intervention and important prognostic factors on the cluster and individual levels (e.g. size of nursing home) and random effects for clusters. The random effects are included to account for possible intra-cluster correlation. If a larger proportion of patients than anticipated should die within 12 months or discontinue the study prematurely, we will use the time to first hospitalisations as endpoint, which will be analysed using a semi-parametric model with proportional hazards, and mixed effects as above. Death and study discontinuation will be dealt with as competing events. The secondary endpoints quality of life and satisfaction of the residents regarding the interaction of the general practitioners and nursing staff will be modelled using hierarchical models with random cluster effects.

The qualitative and quantitative data collected for process evaluation will be analysed in a multistep approach: First, qualitative and quantitative data will be descriptively analysed independently from each other. Then these qualitative and quantitative findings will be cross-mapped for each single research question addressed by the process evaluation. Based on this descriptive synthesis, a regression model will be defined to explore the effects of the degree of intervention implementation and potential moderator and modifier variables on the quality of interprofessional collaboration between medical doctors and nursing homes and its interaction with the primary outcome. The regression model and analysis procedures will be chosen based on the statistical characteristics of relevant variables. A detailed description of the analysis methods is included in the separate protocol for the process evaluation (under submission).

Health economic evaluation will be conducted as cost-effectiveness analysis (outcome incremental cost-effectiveness ratio (ICER) from payer perspectives (statutory health insurance, long-term care insurance), as well as from a societal perspective). To analyse uncertainty regarding the ICER, net-benefit regressions will be conducted controlling for relevant confounders (e.g. baseline values of costs and EQ-5D-5L, cluster structure, morbidity) and cost-effectiveness acceptability curves will be estimated. In addition, a cost-comparison analysis will conducted using difference-in-difference regression to identify relevant cost drivers.

### Data monitoring

The Clinical Trials Unit of the Medical University Center Göttingen, which is independent from the funding organisation, will monitor the Cluster-RCT. A central external audit by the Clinical Study Management will guarantee consistent study procedures within the three research centres. Data monitoring is performed according to a data-monitoring manual following GCP.

### Assessment of risks and benefits

The research study holds no risk for participating NHRs. It is rather expected that all participating NHRs as well as the involved GPs and care staff will benefit from the *interprof* ACT measures. First, positive results have been gathered from interviews after the three-monthly pilot study *interprof* with four nursing homes [31].

Occurring adverse events will be detected, monitored and documented from an early stage onwards. We collect information about the incidence of pressure ulcers, chronic wounds, pneumonia and falls from residents’ files. We plan to publish the findings on these topics together with the main outcome. Meetings and telephone conferences in regular intervals ensure the information and communication processes between the involved research centres.

The planned methods and key recruitment numbers of NHRs are based on previously conducted clinical studies such as several Cluster-RCTs within the setting of long-term inpatient care [47, 52, 54], by the research centres involved. Established co-operations with care facilities and GPs (e.g. research networks), their experience with interprofessional projects such as the *interprof* study [31] and the collaboration with the experts committee contribute to a robust study design.

The study will be planned, implemented and evaluated in accordance with the principles of good clinical practice (ICH-GCP) and the current version of the Declaration of Helsinki.

### Quality assurance

An expert advisory board established for this trial will supervise the trial planning and implementation with specific considerations to the feasibility and practicability of study procedures and interventions to the NHRs, their informal caregivers and the health professionals involved. The members are independent of both investigators and sponsor. The expert committee consists of ten experts representing following perspectives: NHRs, informal caregivers NHR, nursing home advisory boards, science and practice of nursing and geriatric care, GP practice. The integration of this expert committee assures that the results of the *interprof * ACT trial are of high internal validity.

The name of the NHR (and the nursing home) as well as confidential information falls under the medical confidentiality regulation and the regulations of the Federal Data Protection Law (Bundesdatenschutzgesetz BDGS). Data collected in this project will be recorded on paper case forms or electronic data storage, treated in strict confidence and will only be transmitted without mentioning any names (pseudonymised). Access to the original documents will be denied for third parties. The same applies to all additional data collected for the process evaluation. Data can be transmitted to third parties (e.g. journals), but only in a form which does not allow the identification of a person (anonymised). The access to the final dataset will be given from the study statistician.

### Dissemination

Trial results will be disseminated via research articles, contributions on national and international congresses and in local events of the participating institutes. Moreover, we will inform participating nursing homes and GPs about the findings. The results will be disseminated regardless of the magnitude or direction of findings. This study protocol was written according to the SPIRIT Statement.

## Discussion

As NHRs often experience potentially avoidable nursing home admissions, which could have been avoided, the aim of our study is to reduce hospitalisations of NHRs by implementing a systematically developed and pre-tested intervention package addressing the quality of interprofessional collaboration and communication between GPs and nursing homes staff who share the main responsibility for the medical care. The six measures of *interprof* ACT have been developed in the precedent study interprof and included the perspectives of GPs, nurses, residents and relatives on interprofessional collaboration and medical care in nursing homes. One of the developed measures (meetings to establish common goals) even comprises directly the inclusion of resident’s perspective and those of his relatives in the meeting. To improve care, a reduction of the incidence of hospitalisation in NHRs by the *interprof* ACT intervention is expected.

Because of its systematic development and its flexible nature, *interprof* ACT is expected to be viable for standard care regardless of local organisational forms and resources available on a regular basis. Based on the final overall assessment, recommendations will be made for the further design of primary care for NHRs.

In addition, the results may be the starting point for the development of similar strategies for the specialist care of NHRs or interprofessional collaboration in the outpatient care of people in need of care.

In principal, we intend to attract more notice to NHRs and the quality of their healthcare especially in time of demographic change. With the results of this study, we like to spotlight the current situation of NHRs in Germany and contribute to the improvement of their medical care.

## Trial status

Recruitment of NHRs is finished, follow-up ongoing.

## Supplementary information

**Additional file 1.** Spirit Checklist.

**Additional file 2.** Information sheet nursing home resident.

**Additional file 3.** Informed consent form nursing home resident.

**Additional file 4.** Roles and tasks of institutes and persons.

**Additional file 5.** Date and version identifier.

**Additional file 6.** Clinical trials protocol registration.

**Additional file 7.** Team meetings.

## Data Availability

Data are available upon request to the publication committee of the interprof ACT project group.

## Abbreviations

NHR: Nursing home resident
GP: General practitioner
Cluster-RCT: Cluster randomised controlled trial
SGB: Social code book
ICH-GCP: International Conference on Harmonisation - Good Clinical Practice
GDPR: General Data Protection regulation
UMG: University Medical Center Göttingen
FIMA: Questionnaire for Health-Related Resource Use in an Elderly
PRISCUS: Potentially inappropriate medication for older people
QoL-AD NH: Quality of life-Alzheimer’s disease questionnaire - nursing home version
EQ-5D-5L: Standardised instrument developed by the EuroQol Group as a measure of health-related quality of life
DSS: Dementia Screening Scale
QALYs: Quality-adjusted life years
ICC: Intra-cluster correlation
ITT: Intention to treat
GLMM: Generalised linear mixed effects model
ICER: Incremental cost-effectiveness ratio
BDGS: Federal Data Protection Law
CONSORT: Consolidated Statement of Reporting Trials
TIDieR: Template for Intervention Description and Replication
t0: Baseline data collection
t1: Evaluation after 6 months
t2: Evaluation after 12 months
