# Supplementary material for: Effects of strategies to improve general practitioner-nurse collaboration and communication in regard to hospital admissions of nursing home residents (interprof ACT): study protocol for a cluster randomised controlled trial
Source: Trials. 2020 Nov 5;21:913. doi: 10.1186/s13063-020-04736-x (PMC7643262; doi:10.1186/s13063-020-04736-x)
Supplement: Supplementary file 1 — Additional file 1. Spirit Checklist. [file 13063_2020_4736_MOESM1_ESM.docx]

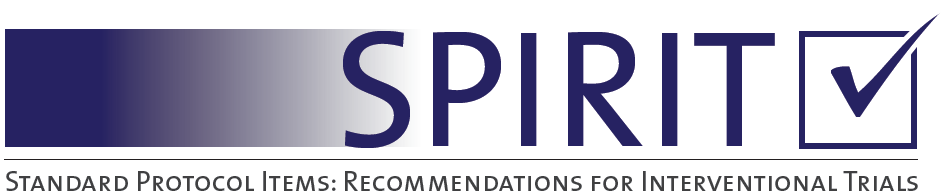


SPIRIT 2013 Checklist: Recommended items to address in a clinical trial protocol and related documents*

| Section/item | Item No | Description |  |
| --- | --- | --- | --- |
| **Administrative information** | | |  |
| Title | 1 | Descriptive title identifying the study design, population, interventions, and, if applicable, trial acronym  All information is included in the title, page 1 line 2-4  Abstract title, page 2 line 42-44 |  |
| Trial registration | 2a | Trial identifier and registry name. If not yet registered, name of intended registry  Page 2 line 86 |  |
|  | 2b | All items from the World Health Organization Trial Registration Data Set  Document is uploaded |  |
| Protocol version | 3 | Date and version identifier  Document is uploaded |  |
| Funding | 4 | Sources and types of financial, material, and other support  Page 22 line 596-605 |  |
| Roles and responsibilities | 5a | Names, affiliations, and roles of protocol contributors  Page 23 line 60-612 |  |
|  | 5b | Name and contact information for the trial sponsor  Page 22 line 600-601. |  |
|  | 5c | Role of study sponsor and funders, if any, in study design; collection, management, analysis, and interpretation of data; writing of the report; and the decision to submit the report for publication, including whether they will have ultimate authority over any of these activities  Page 22 line 602-605 |  |
|  | 5d | Composition, roles, and responsibilities of the coordinating centre, steering committee, endpoint adjudication committee, data management team, and other individuals or groups overseeing the trial, if applicable (see Item 21a for data monitoring committee)  Document “roles and tasks of institutes and persons” is uploaded |  |
| Introduction |  |  |  |
| Background and rationale | 6a | Description of research question and justification for undertaking the trial, including summary of relevant studies (published and unpublished) examining benefits and harms for each intervention  Page 3-5 line 90-163 |  |
|  | 6b | Explanation for choice of comparators  Page 5 line 169 |  |
| Objectives | 7 | Specific objectives or hypotheses  Page 5-6, line 164-178 |  |
| Trial design | 8 | Description of trial design including type of trial (eg, parallel group, crossover, factorial, single group), allocation ratio, and framework (eg, superiority, equivalence, noninferiority, exploratory)  Page 6 line 180-187 in Methods as recommended in the study protocol guidelines of Trials  Figure 1 |  |
| Methods: Participants, interventions, and outcomes | | |  |
| Study setting | 9 | Description of study settings (eg, community clinic, academic hospital) and list of countries where data will be collected. Reference to where list of study sites can be obtained  Data is collected in Germany. Study sites are the three study centers in Göttingen, Hamburg and Lübeck, names of participating nursing homes will not be presented.  Page 6 line 182 |  |
| Eligibility criteria | 10 | Inclusion and exclusion criteria for participants. If applicable, eligibility criteria for study centres and individuals who will perform the interventions (eg, surgeons, psychotherapists)  Inclusion and exclusion criteria for nursing homes: Page 6-7 line 190-199.  Inclusion and exclusion criteria for nursing home residents in Table 1 |  |
| Interventions | 11a | Interventions for each group with sufficient detail to allow replication, including how and when they will be administered  Page 8-10 line 227-293  As our study is not a medical product or a medical devices trial but a complex intervention study in primary care, more detailed information about the implementation could not be provided here. |  |
|  | 11b | Criteria for discontinuing or modifying allocated interventions for a given trial participant (eg, drug dose change in response to harms, participant request, or improving/worsening disease)  Not applicable  Not applicable.  It is part of the study that the intervention is tailored to each participating nursing home of the intervention group according to the results of the Kick-off meeting. Moreover, modifications during the implementation or follow up are allowed and are monitored regularly in the supervision contacts. More details will be provided in the protocol of the process evaluation. |  |
|  | 11c | Strategies to improve adherence to intervention protocols, and any procedures for monitoring adherence (eg, drug tablet return, laboratory tests)  Strategies to improve adherence are regular supervision contacts by the local research team: Page 9-10 line 268-279, moreover nursing homes and GPs of the intervention group receive 150 Euro per patient, of the control group 50 Euro per patient expenditure allowance: Page 8 line 223-225. |  |
|  | 11d | Relevant concomitant care and interventions that are permitted or prohibited during the trial  Not applicable  All necessary care or interventions are permitted at the discretion of treating physicians or nurses.  Prohibited: Participation in another trial concerning interprofessional collaboration. |  |
| Outcomes | 12 | Primary, secondary, and other outcomes, including the specific measurement variable (eg, systolic blood pressure), analysis metric (eg, change from baseline, final value, time to event), method of aggregation (eg, median, proportion), and time point for each outcome. Explanation of the clinical relevance of chosen efficacy and harm outcomes is strongly recommended  Outcomes: Page 11-13 line 302-370  Table 3 and Table 4 |  |
| Participant timeline | 13 | Time schedule of enrolment, interventions (including any run-ins and washouts), assessments, and visits for participants. A schematic diagram is highly recommended (see Figure)  Table 2 |  |
| Sample size | 14 | Estimated number of participants needed to achieve study objectives and how it was determined, including clinical and statistical assumptions supporting any sample size calculations  Page 14 line 389-407 |  |
| Recruitment | 15 | Strategies for achieving adequate participant enrolment to reach target sample size  Page 7-8 line 203-225 |  |
| **Methods: Assignment of interventions (for controlled trials)** | | |  |
| Allocation: |  |  |  |
| Sequence generation | 16a | Method of generating the allocation sequence (eg, computer-generated random numbers), and list of any factors for stratification. To reduce predictability of a random sequence, details of any planned restriction (eg, blocking) should be provided in a separate document that is unavailable to those who enrol participants or assign interventions  Page 15 line 409-417 |  |
| Allocation concealment mechanism | 16b | Mechanism of implementing the allocation sequence (eg, central telephone; sequentially numbered, opaque, sealed envelopes), describing any steps to conceal the sequence until interventions are assigned  Page 15 line 418-420 |  |
| Implemen-tation | 16c | Who will generate the allocation sequence, who will enrol participants, and who will assign participants to interventions  The allocation sequence is generated by the Department of Medical Statistics UMG (Page 15 line 410-412), research team members of each centre will enrol nursing homes and NHR (Page 7 line 204 and 208-214), the trial statistician will assign nursing homes to interventions (Page 15 line 407-409). |  |
| Blinding (masking) | 17a | Who will be blinded after assignment to interventions (eg, trial participants, care providers, outcome assessors, data analysts), and how  Page 15 line 421-430 |  |
|  | 17b | If blinded, circumstances under which unblinding is permissible, and procedure for revealing a participant’s allocated intervention during the trial  Not applicable as all nursing homes and nursing home residents, GPs and researcher except those collecting information on the primary outcome are not blinded to the allocation. |  |
| **Methods: Data collection, management, and analysis** | | |  |
| Data collection methods | 18a | Plans for assessment and collection of outcome, baseline, and other trial data, including any related processes to promote data quality (eg, duplicate measurements, training of assessors) and a description of study instruments (eg, questionnaires, laboratory tests) along with their reliability and validity, if known. Reference to where data collection forms can be found, if not in the protocol  Our main outcome is the proportion incidence of hospital admissions. The secondary outcomes contain some data collection instruments, which are validated: Questionnaire for Health-Related Resource Use in an Elderly, the Quality of life-Alzheimer’s disease questionnaire (QoL-AD NH), the standardized instrument as a measure of health-related quality of life (EQ-5D-5L). Depending on the NHRs’ cognition, either self-reported judgements by the NHRs or proxy judgements by nurses will be retrieved for this quality of life assessment. To appraise residents’ ability to answer questionnaires and as an indication of cognitive impairments the Dementia Screening Scale (DSS) will be applied as proxy measure by nursing home staff.  Validity of instruments is provided Page 11-12 line 302-344 |  |
|  | 18b | Plans to promote participant retention and complete follow-up, including list of any outcome data to be collected for participants who discontinue or deviate from intervention protocols  Nursing home residents receive a small thank you gift for participation, GPs and nursing home an expenditure allowance for each participant at the end of the study.  Page 8 line 223-225.  Handling of leaving or dying participants: Page 14 line 394. |  |
| Data management | 19 | Plans for data entry, coding, security, and storage, including any related processes to promote data quality (eg, double data entry; range checks for data values). Reference to where details of data management procedures can be found, if not in the protocol  Page 16 line 431-441  Page 19 line 509-517  Details of data management procedures can be found in our SOPs of data collection and data entry. A copy of those SOPs is also stored in the Clinical Trials Unit of UMG. |  |
| Statistical methods | 20a | Statistical methods for analysing primary and secondary outcomes. Reference to where other details of the statistical analysis plan can be found, if not in the protocol  Page 16-17 line 442-456  More details can be found in a statistical analyses plan. |  |
|  | 20b | Methods for any additional analyses (eg, subgroup and adjusted analyses)  Page 17 line 457-475  More details will be found in the protocol of the process evaluation |  |
|  | 20c | Definition of analysis population relating to protocol non-adherence (eg, as randomised analysis), and any statistical methods to handle missing data (eg, multiple imputation)  Page 16 line 442-456 |  |
| **Methods: Monitoring** | | |  |
| Data monitoring | 21a | Composition of data monitoring committee (DMC); summary of its role and reporting structure; statement of whether it is independent from the sponsor and competing interests; and reference to where further details about its charter can be found, if not in the protocol. Alternatively, an explanation of why a DMC is not needed  Data will be monitored by the Clinical Trials Unit of UMG, which is independent from the funder. It belongs to UMG, which is the sponsor but acts independently from the Institute of General Practice.  Page 17 line 476-480 |  |
|  | 21b | Description of any interim analyses and stopping guidelines, including who will have access to these interim results and make the final decision to terminate the trial  A sample size review is performed after 300 recruited nursing home residents to adjust the sample size.  Page 15 line 404-407 |  |
| Harms | 22 | Plans for collecting, assessing, reporting, and managing solicited and spontaneously reported adverse events and other unintended effects of trial interventions or trial conduct  Page 18 line 487-491 |  |
| Auditing | 23 | Frequency and procedures for auditing trial conduct, if any, and whether the process will be independent from investigators and the sponsor  Page 18-19 line 500-508 (Advisory Board)  Extra document on team meetings uploaded |  |
| Ethics and dissemination | | |  |
| Research ethics approval | 24 | Plans for seeking research ethics committee/institutional review board (REC/IRB) approval  Page 21 line 561-568  Ethical approvals of all centres are uploaded |  |
| Protocol amendments | 25 | *Plans for communicating important protocol modifications (eg, changes to eligibility criteria, outcomes, analyses) to relevant parties (eg, investigators, REC/IRBs, trial participants, trial registries, journals, regulators)*  Page 21 line 565-571  Moreover important changes of the protocol are communicated to the funder and have to be agreed on. Modifications of the intervention are expected to be part of the implementation process, and are consented and communicated during the kick-off-meetings. |  |
| Consent or assent | 26a | Who will obtain informed consent or assent from potential trial participants or authorised surrogates, and how (see Item 32)  Page 7 line 208--215  Page 21 line 572-587 |  |
|  | 26b | Additional consent provisions for collection and use of participant data and biological specimens in ancillary studies, if applicable  Not applicable as no ancillary studies exist. |  |
| Confidentiality | 27 | How personal information about potential and enrolled participants will be collected, shared, and maintained in order to protect confidentiality before, during, and after the trial  Page 19 line 509-517 |  |
| Declaration of interests | 28 | Financial and other competing interests for principal investigators for the overall trial and each study site  Page 22 line 594 |  |
| Access to data | 29 | Statement of who will have access to the final trial dataset, and disclosure of contractual agreements that limit such access for investigators  The protocol should identify the individuals involved in the trial who will have access to the full dataset. Any restrictions in access for trial investigators should also be explicitly described.  Data are available upon request to the publication committee of the interprof ACT project group. |  |
| Ancillary and post-trial care | 30 | Provisions, if any, for ancillary and post-trial care, and for compensation to those who suffer harm from trial participation  Page 11 line 300-301 |  |
| Dissemination policy | 31a | Plans for investigators and sponsor to communicate trial results to participants, healthcare professionals, the public, and other relevant groups (eg, via publication, reporting in results databases, or other data sharing arrangements), including any publication restrictions  Page 19 line 518-523  Statement of publication regardless of effect  Page 19 line 521-522 |  |
|  | 31b | Authorship eligibility guidelines and any intended use of professional writers  Authorship will be determined according to the Recommendations for the Conduct, Reporting, Editing, and Publication of Scholarly work in Medical Journals of the international committee of the medical journal editors (<http://www.icmje.org/recommendations/>) Submitting institutes have to follow recommendations, the publication committee will control if content and authorships are adequate.  No professional writers were involved. |  |
|  | 31c | Plans, if any, for granting public access to the full protocol, participant-level dataset, and statistical code  Protocol and results should be published open source. |  |
| Appendices |  |  |  |
| Informed consent materials | 32 | Model consent form and other related documentation given to participants and authorised surrogates  Form is included in additional material |  |
| Biological specimens | 33 | Plans for collection, laboratory evaluation, and storage of biological specimens for genetic or molecular analysis in the current trial and for future use in ancillary studies, if applicable  Not applicable: no biological specimens will be collected as part of this trial |  |

*It is strongly recommended that this checklist be read in conjunction with the SPIRIT 2013 Explanation & Elaboration for important clarification on the items. Amendments to the protocol should be tracked and dated. The SPIRIT checklist is copyrighted by the SPIRIT Group under the Creative Commons “[Attribution-NonCommercial-NoDerivs 3.0 Unported](http://www.creativecommons.org/licenses/by-nc-nd/3.0/)” license.
