# Supplementary material for: Effects of strategies to improve general practitioner-nurse collaboration and communication in regard to hospital admissions of nursing home residents (interprof ACT): study protocol for a cluster randomised controlled trial
Source: Trials. 2020 Nov 5;21:913. doi: 10.1186/s13063-020-04736-x (PMC7643262; doi:10.1186/s13063-020-04736-x)
Supplement: Supplementary file 2 — Additional file 2. Information sheet nursing home resident. [file 13063_2020_4736_MOESM2_ESM.pdf]

Prof. Dr. med. Eva Hummers
Institut für Allgemeinmedizin
Universitätsmedizin Göttingen
Humboldtallee 38
D-37073 Göttingen

UNIVERSITÄTSMEDIZIN GÖTTINGEN UMG
ALLGEMEINMEDIZIN

Information für Bewohnerinnen und Bewohner

Studie: „Auswirkungen von Maßnahmen zur Verbesserung ärztlich-pflegerischer Zusammenarbeit auf Krankenhausaufnahmen von Pflegeheimbewohnern"

Sehr geehrte Dame, sehr geehrter Herr,

wir möchten Sie einladen, an einer Studie teilzunehmen, die das Institut für Allgemeinmedizin der Universitätsmedizin Göttingen gemeinsam mit der Georg-August-Universität Göttingen, dem Universitätsklinikum Hamburg-Eppendorf und der Universität zu Lübeck durchführt.

Bitte lesen Sie diese Information sorgfältig durch.

Warum wird diese Studie durchgeführt?

Ziel dieser Studie ist es, die Zusammenarbeit zwischen Hausärztinnen und Hausärzten und Pflegekräften in Pflegeeinrichtungen zu fördern. Wir möchten in drei Regionen Deutschlands prüfen, ob durch das Einführen von verschiedenen Maßnahmen die ärztlich-pflegerische Zusammenarbeit verbessert werden kann. Wir erwarten, dass es hierdurch u.a. zu weniger Krankenhausaufnahmen von Bewohnerinnen und Bewohnern sowie zur Steigerung der Lebensqualität und Zufriedenheit kommt.

Wie ist der Ablauf der Studie?

Alle teilnehmenden Bewohnerinnen und Bewohner erhalten die gängige und bewährte Betreuung. Ihre medizinische Versorgung durch das Pflegepersonal und Ihren Hausarzt erfolgt in gewohnter Weise.

In der Hälfte der Einrichtungen werden zusätzlich Maßnahmen zur besseren ärztlich-pflegerischen Zusammenarbeit eingeführt. Die Einteilung der teilnehmenden Pflegeeinrichtungen in die Gruppe mit oder ohne diese zusätzlichen Maßnahmen erfolgt per Zufallsprinzip (einem Verteilungsschema, das vor Studienbeginn festgelegt wird). Dies hat den Zweck eine möglichst hohe wissenschaftliche Aussagekraft der Studie zu erreichen.

Die Studie dauert in Ihrer Einrichtung insgesamt 12 Monate. Mit Ihrer Einwilligung werden wir halbjährlich Einsicht in Ihre Pflegedokumentation nehmen, um dort Angaben zu Ihrer pflegerischen und medizinischen Versorgung und Entlassungsdiagnosen aus einem evtl. aufgetretenen Krankenhausaufenthalt zu entnehmen. Zu Beginn und zum Studienende nach einem Jahr bitten wir

Sie, soweit das für Sie möglich ist, mit uns gemeinsam drei kurze Fragebögen zu Ihrer aktuellen Situation auszufüllen (Zeitaufwand ca. 15-20 Minuten). Dabei geht es um Ihre Lebensqualität und die Zufriedenheit mit der medizinischen Versorgung. Außerdem werden wir in regelmäßigen Abständen Ihre Pflegepersonen zur ärztlich-pflegerischen Zusammenarbeit befragen. Ihre Einwilligung in diese Studie vorausgesetzt, werden wir auch Ihre Hausärztin/Ihren Hausarzt über Ihre Teilnahme informieren und zur Zusammenarbeit mit der Pflegeeinrichtung befragen.

Bei der Teilnahme werden keine Risiken oder Schäden erwartet. Im Gegenteil, es wird ein Nutzen durch die Verbesserung der Zusammenarbeit zwischen Pflegekräften und Hausärzten erwartet.

Was geschieht mit meinen Angaben?

Während der Studie werden medizinische Befunde und persönliche Informationen von Ihnen erhoben und in Papierform und auf elektronischen Datenträgern pseudonymisiert (verschlüsselt) gespeichert. Pseudonymisierung bedeutet, dass Nummern oder Buchstabencodes verwendet werden, ggf. in Verbindung mit einem Geburtsjahr. Die für die Studie wichtigen Daten werden zusätzlich in pseudonymisierter Form gespeichert, ausgewertet und gegebenenfalls weitergegeben. Eine nachträgliche Zuordnung der Daten zu einer bestimmten Person ist mit Hilfe einer Art „Schlüssel“, der in der Studienzentrale verwaltet wird, möglich. Die Ergebnisse der Studie werden in anonymisierter Form veröffentlicht (d.h. ein Bezug zu Ihrer Person ist dann nicht mehr möglich). Wir sichern Ihnen zu, dass alle beteiligten Personen die Bestimmungen des Datenschutzgesetzes und die berufliche Schweigepflicht einhalten werden.

Ihre Teilnahme am Forschungsprojekt ist freiwillig. Sie können Ihr Einverständnis jederzeit, ohne Angabe von Gründen und ohne Nachteile für Ihre weitere medizinische und pflegerische Versorgung zurückziehen. Bei Rücktritt von der Studie kann auf Wunsch bereits gewonnenes Datenmaterial vernichtet werden. Sie können sich bei der Rücknahme der Einwilligung entscheiden, ob Sie mit der Auswertung Ihrer Studiendaten einverstanden sind oder nicht. Sollten Sie zu einem späteren Zeitpunkt Ihre Entscheidung ändern wollen, setzen Sie sich bitte mit einem Studienmitarbeiter/-in in Verbindung.

Wer ist bei Fragen ansprechbar?

Wir stehen Ihnen jederzeit gerne für Rückfragen zur Verfügung. Bitte wenden Sie sich an:

Dr. Christiane Müller, 0551-39-22691, christiane.mueller@med.uni-goettingen.de
Dr. Anke Theuerkauf, 0551-39-65575, anke.theuerkauf@med.uni-goettingen.de

Wenn Sie teilnehmen möchten, bestätigen Sie Ihre Einwilligung bitte auf dem beiliegenden Blatt (Einwilligungserklärung) durch Ihre Unterschrift.

Herzlichen Dank für Ihre Unterstützung!

Die Studie wird gefördert vom Gemeinsamen Bundesausschuss.
