# Supplementary material for: Effects of strategies to improve general practitioner-nurse collaboration and communication in regard to hospital admissions of nursing home residents (interprof ACT): study protocol for a cluster randomised controlled trial
Source: Trials. 2020 Nov 5;21:913. doi: 10.1186/s13063-020-04736-x (PMC7643262; doi:10.1186/s13063-020-04736-x)
Supplement: Supplementary file 3 — Additional file 3. Informed consent form nursing home resident. [file 13063_2020_4736_MOESM3_ESM.pdf]

Prof. Dr. med. Eva Hummers
Institut für Allgemeinmedizin
Universitätsmedizin Göttingen
Humboldtallee 38
D-37073 Göttingen

interprof ACT

UNIVERSITÄTSMEDIZIN GÖTTINGEN UMG
ALLGEMEINMEDIZIN

Für Ihre Unterlagen

Einverständniserklärung zur Teilnahme an der Studie

„Auswirkungen von Maßnahmen zur Verbesserung ärztlich-pflegerischer Zusamme[illegible] Krankenhausaufnahmen von Pflegeheimbewohnern (*interprof* ACT)“

Ich wurde über die o. g. Studie von einem Studienmitarbeiter mündlich informiert und habe das Informationsschreiben erhalten, gelesen und verstanden. Ich hatte die Gelegenheit, Fragen dazu zu stellen, die zu meiner Zufriedenheit beantwortet wurden.

Ich hatte genügend Zeit, mich zur Teilnahme zu entscheiden. Ich weiß, dass die Teilnahme freiwillig und mit keinem unmittelbaren persönlichen Nutzen oder Nachteilen für verbunden ist. Ich weiß, dass ich diese Zustimmung jederzeit widerrufen kann, ohne dass dadurch Nachteile für mich oder die Einrichtung entstehen.

Ich bin einverstanden, dass im Rahmen dieses Forschungsvorhabens personenbezogene Daten erhoben und in pseudonymisierter (verschlüsselter) Form aufgezeichnet und gespeichert werden. Die Datenspeicherzeit beträgt 10 Jahre. Die personenbezogenen Daten werden nicht an Dritte weitergegeben. Ich bin einverstanden, dass die pseudonymisierten Daten an Dritte weitergegeben werden können, jedoch nur in einer Form, die keine Rückschlüsse auf Personen zulässt. Die personenbezogenen Daten bzw. der Pseudonymisierungsschlüssel werden unter Verschluss gehalten; sie sind nur der Projektleiterin und ihrer Vertretung zugänglich. Die pseudonymisierten Daten werden von Projektmitarbeitern ausgewertet und für wissenschaftliche Veröffentlichungen und die medizinische Lehre genutzt.

Ich weiß, dass ich mein Einverständnis zur Speicherung der personenbezogenen Daten jederzeit widerrufen kann. Im Falle des Widerrufs werden alle personenbezogenen Daten gelöscht.

Ich habe den Informationstext und eine Kopie dieser Einwilligung erhalten.

Zudem habe ich die „Ergänzende Information zur aktualisierten Datenschutzgrundverordnung (DSGVO)“ erhalten und wurde über die Inhalte mündlich aufgeklärt.

Hiermit erkläre ich meine freiwillige Einwilligung zur Teilnahme an der Studie.

______________________________ ______________________________

Vor- und Nachname des Bewohners/der Bewohnerin — Ort, Datum, Unterschrift Bewohner/ Bewohnerin

____

Ort, Datum, Unterschrift Studienmitarbeiter
