# Supplementary material for: Effects of strategies to improve general practitioner-nurse collaboration and communication in regard to hospital admissions of nursing home residents (interprof ACT): study protocol for a cluster randomised controlled trial
Source: Trials. 2020 Nov 5;21:913. doi: 10.1186/s13063-020-04736-x (PMC7643262; doi:10.1186/s13063-020-04736-x)
Supplement: Supplementary file 4 — Additional file 4. Roles and tasks of institutes and persons. [file 13063_2020_4736_MOESM4_ESM.docx]

**4.1 Organisational structure and responsibilities**

**Study Center Göttingen**

**University Medical Center Göttingen,**

**Institute of General Practice**

(Prof. Dr. med. Eva Hummers (Principal Investigator), Dr. med. Christiane Müller, MPH* and research team: Vivien Weiß, Berit Hesjedal- Streller, Maria Hendrich, Caroline Birau, study assistant: Anke Theuerkauf)

- Design and conduct of interprof ACT
- Preparation of study documents and revisions
- Preparation of CRFs [Case Report Forms]
- Organisation of steering committee meetings
- Recruitment of participants (nursing homes, residents, general practitioners, registered nurses)
- Involvement in preparation of trial IT system for interprof ACT (SecuTrail^®^)
- Reviewing progress of study and if necessary, agreeing changes to the protocol and/or investigators brochure to facilitate the smooth running of the study
- Publication of study reports

**Georg August University Göttingen.**

**Chair of Organization and Corporate Development**

(Prof. Dr. Indre Maurer and research team: Dr. Clarissa Weber*, Christian Kortkamp)

- Design and conduct of interprof ACT
- Conduct of qualitative process evaluation
- Design and preparation of documents and revisions for qualitative process evaluation

**Study Center Hamburg**

**University Medical Center Hamburg-Eppendorf**

**Department of General Practice/Primary Care**

(Prof. Dr. med. Martin Scherer and research team: Dr. Britta Tetzlaff*, Tina Mallon*, Annette Strauß, Dr. Thomas Zimmermann, Gerrit von Deyen, study assistants: Heike Kretzschmer)

- Design and conduct of interprof ACT
- Preparation of documents and revisions
  Preparation of CRFs [Case Report Forms]
  Recruitment of participants (nursing homes, residents, general practitioners)
- Conduct of quantitative process evaluation

**Department of Health Economics and Health Services Research**

(Prof. Hans-Helmut König, PD Dr. Alexander Konnopka*)

- Design and conduct of interprof ACT
- Conduct of health economic evaluation

**Study Center Lübeck**

**University of Lübeck**

**Institute for Social Medicine and Epidemiology, Nursing Research Group**

(Prof. Dr. phil. Sascha Köpke, Prof. Dr. rer. cur. Katrin Balzer and research team: Anja Behncke*, Linda Gärtner*, study assistants: Frederike Lüth, Anne-Marei Jarchow)

- Design and conduct of interprofACT
- Preparation of study documents and revisions
  Preparation of CRFs [Case Report Forms]
  Recruitment of participants (nursing homes, residents, general practitioners)
- Design and conduct of quantitative process evaluation
- Preparation of documents and revisions for quantitative process evaluation (incl. pretest)
- Involvement in preparation of trial IT system for interprof ACT process evaluation (SecuTrail^®^)
- Organisation and communication with Advisory Board

**Trial project monitoring**

**University Medical Center Göttingen**

**Clinical Trails Unit**

(Nadine Röthling, Harald Müller, Christopher Schuchardt, Nicole Kirchhof)

- Advice for lead investigators
- Monitoring of t0 data collection and t2 data collection

**Data management**

**Clinical Trials Unit, University Medical Center Göttingen**

(Tina Krüger, Sebastian Pfeiffer, Aaron Marshall)

- Setoff of data IT system (SecuTrial)
- Maintenance of trial IT system and data entry
- Data verification

**Trial statistician**

**Department of Medical Statistics, University Medical Center Göttingen**

(Prof. Tim Friede, PhD, Sebastian Pfeiffer)

- Design of interprof ACT
- Sample size calculation
- Randomisation
- Data analyses

***Lead Investigators**In each participating centre a lead investigator will be identified, to be responsible for identification, recruitment, data collection and completion of CRFs, along with follow up of study patients and adherence to study protocol and investigators brochure
