# Supplementary material for: Effects of strategies to improve general practitioner-nurse collaboration and communication in regard to hospital admissions of nursing home residents (interprof ACT): study protocol for a cluster randomised controlled trial
Source: Trials. 2020 Nov 5;21:913. doi: 10.1186/s13063-020-04736-x (PMC7643262; doi:10.1186/s13063-020-04736-x)
Supplement: Supplementary file 5 — Additional file 5. Date and version identifier. [file 13063_2020_4736_MOESM5_ESM.docx]

Date and version identifier study protocol interprof ACT

| Date of amendment submission | Topics |
| --- | --- |
| 1. Amendment 15.08.2017 | - Inclusion of interprof ACT measures into information sheet for nursing home (NH). |
| 1. Amendment 06.11.2017 | - Update of research team members in study protocol - Inclusion of suggestions for the use of the expenditure allowance for nursing homes in new document for nursing homes - Informed consent collected also by research team members, not only physicians - Hospital discharge diagnosis to be photograped, information added in study protocol and in information sheet nursing home resident (NHR) - Relatives will be included into shared goal setting and kick-off meetings - NHRs` and relatives` information about allocation of nursing home - Information on early drop out of residents   New documents   - Extra documents for inclusion and exclusion criteria of nursing homes and NHRs - Inclusion of expected expenditure of time for GPs in information sheet - Process evaluation: All information sheets, informed consent forms, questionnaires, guidelines for interviews and non- participating observations |
| 1. Amendment 08.02.2018 | - No new content, only adjustment of phrases within the study protocol |
| 1. Amendment 13.03.2018 | - Adjustment of phrases within the studyprotocol, change of GP visit to GP contact with regard to inclusion criteria NHR - More emphasis on the recruitment of GPs ahead of randomisation - Addition of columns minimal application frequency and max. single dose an a new column for own remarks. Moreover indication and strength oft the medication were added. - Small changes in NH demography questionnaire - Small changes in NHR demography questionnaire - Addition of possibity to choose „answer not possible“ to some items of main questionnaire plus some formal remarks - Addition of the possibity to choose „assessment of heath today not possible“ to the items of EQ5D and „ cannot be assessed „ in the quantitative process evaluation (NHR quality of life, satisfaction with medical procedures). Small changes in the wording of the questionnaire. |
| 1. Amendment 18.04.2018 | - Inclusion of Information according tot he new data protection law into the informed consent forms - Practice nurses are invited to kick-off meetings, too - GPs of control group recieve the information sheet benefits of good interprofessional collaboration - Dementia screening scale will be also used at T2 data collection - EQ5D: Assessment by Betreuer deleted - Person, who gives information, signs form about inclusion and exclusion criteria - Expenditure allowance will be paid for GPs at the end of the follow-up time - Small changes of wording process evaluation (GPs`and nurses`questionnaires, questionnaire about training of interprof ACT agents, before and after questionnaire of kick-of meeting,)   New documents process evaluation:   - 2. training of interprof ACT agent - Protocol 1. Training interprof ACT agent - Protocol 2. Training interprof ACT agent - Information on trainers of interprof ACT agent trainings - Protocol kick-off meeting - Protocol supervision face to face contacts - Protocol supervision via telephone |
| 1. Amendment 08.08.2018 | - Update of research team members in study protocol - Legal representative receives written information material, oral information by research team via telephone is also allowed - Increase of recruited NH for process evaluation - Small changes in protocol supervision - Changes of contact person in study documents   New documents   - Information on new data protection law for NHR, legal representative and NH management |
| 1. Amendment 05.04.19 | - Change of contact persons - CRF: Some formatting changes, GP contacs with number of faxes (new), chronic wounds not during last 6 months but with date of prevalence.   New documents   - Demography nursing home t2 - Not finished hospital stay (supporting document for data collectors) - Process evaluation: questionnaires GPs and nurses t2, questionnaires for interprof ACT agents t1 and t2 |
| 1. Amendment 24.06.2019 | - Update of research team members - Change of criteria for DSS t2: if Score is 4 (and not only less than 4) NHR can answer the following questionnaires, if he likes to. If NHR declines, the nurse will be asked to answer the questionnaires. Missing items in DSS will be considered deciding about the person to answer the following questions. |
| 1. Amendment 16.12.2019 | - Update of research team members - Change of criteria for DSS t2: If score is 4 (and not only less than 4) NHR can answer the following questionnaires, if he likes to. If NHR declines, also the nurse will not be asked to answer the questionnaires. Missing items in DSS will be considered deciding about the person to answer the following questions. - The six measures will be also implemented into NH of control group, if they want to. Interprof ACT agents will be trained by resarch team. Nursing homes will contact GPs on their own and will also organise the kick-off meeting on their own. No regular supervision by research team is planned. The implementation will be evaluated in a quantitative process evaluation. - Small changes in questionnaire GP process evaluation t2 - Small changes in supervision document (examples added) - Small changes questionnaire interprof ACT agent process evaluation t2   New documents   - Expenditure sheets for reimbursement GPs and NH - Protocol for training of new interprof ACT agents during the follow up - Protocol of change of interprof ACT agent and questionnaire for interprof ACT agents about change - Questionnaire regarding blinding for research team members - All documents for process evaluation implementation of measures in NH of control group. |
