# Supplementary material for: Effects of strategies to improve general practitioner-nurse collaboration and communication in regard to hospital admissions of nursing home residents (interprof ACT): study protocol for a cluster randomised controlled trial
Source: Trials. 2020 Nov 5;21:913. doi: 10.1186/s13063-020-04736-x (PMC7643262; doi:10.1186/s13063-020-04736-x)
Supplement: Supplementary file 6 — Additional file 6. Clinical trials protocol registration. [file 13063_2020_4736_MOESM6_ESM.pdf]

ClinicalTrials.gov PRS
Protocol Registration and Results System

ClinicalTrials.gov Protocol Registration and Results System (PRS) Receipt
Release Date: February 7, 2018

ClinicalTrials.gov ID: NCT03426475

Study Identification

Unique Protocol ID: 01811

Brief Title: Effect of Strategies to Improve General Practitioner-nurse Collaboration and Communication (interprof ACT)

Official Title: Effects of Strategies to Improve General Practitioner-nurse Collaboration and Communication in Regard to Hospital Admissions of Nursing Home Residents.

Secondary IDs:

Study Status

Record Verification: February 2018

Overall Status: Not yet recruiting

Study Start: February 2018 [Anticipated]

Primary Completion: December 2019 [Anticipated]

Study Completion: March 2020 [Anticipated]

Sponsor/Collaborators

Sponsor: University Medical Center Goettingen

Responsible Party: Principal Investigator
Investigator: Karsten Gavenis [kgavenis]
Official Title: Clinical Study Management on behalf of the Principal Investigator
Affiliation: University Medical Center Goettingen

Collaborators:

Oversight

U.S. FDA-regulated Drug: No

U.S. FDA-regulated Device: No

U.S. FDA IND/IDE: No

Human Subjects Review: Board Status: Pending
Board Name: Ethics Committee
Board Affiliation: Ethics Committee Goettingen University Medical Center
Phone: +49 551 3968644
Email: ethik@med.uni-goettingen.de
Address:

Von-Siebold-Str. 3
37075 Goettingen
Germany

Data Monitoring: No

FDA Regulated Intervention: No

Study Description

Brief Summary: Previously,six measures were developed for a better collaboration of general practitioners and nurses in nursing homes in a qualitative multistep bottom-up process. These measures, summarised as the interprof ACT intervention, shall improve the flow of information and the communication between the involved parties and lead to more transparency and effectiveness regarding treatment decisions of nursing home residents.The major aim of this trial is to examine the clinical effectiveness of interprof ACT. The main hypothesis is that implementation of interprof ACT reduces the cumulative incidence of hospitalisations of nursing home residents within 12 months from 50% to 35% (15% absolute reduction).

Detailed Description:

Conditions

Conditions: Utilization of Medical Care by Nursing Home Residents

Keywords: nursing home residents
interprofessional collaboration
reduction of hospitalisation

Study Design

Study Type: Interventional

Primary Purpose: Supportive Care

Study Phase: N/A

Interventional Study Model: Parallel Assignment

Number of Arms: 2

Masking: None (Open Label)
Blinded study nurses with respect of primary outcome.

Allocation: Randomized

Enrollment: 680 [Anticipated]

Arms and Interventions

Arms	Assigned Interventions
No Intervention: Control Group<br>Care of nursing home residents as usual.	
Experimental: Interventional Group<br>Implementation of interprof ACT measures to improve collaboration and communication between general practitioners and nursing staff. Measures are selected and adapted by nursing home management / nurses, GPs and residents' relatives or representatives.	Procedure/Surgery: interprof ACT measures<br>Definition of common goals between general practitioner and nursing staff, appointment of a contact person, support in assigning medication, use of name badges worn by GPs and nurses during visits,

Arms	Assigned Interventions
	mandatory availability of contact person, standardized procedures for GPs home visits

Outcome Measures

Primary Outcome Measure:

1. cumulative number of hospitalisation
   Cumulative incidence of hospitalisations from nursing home residents within 12 months

   [Time Frame: within 12 months]

Secondary Outcome Measure:

2. absolute number of hospitalisations
   number of hospitalisations within 12 months

   [Time Frame: within 12 months]
3. hospital days
   Hospital days within 12 months

   [Time Frame: within 12 months]
4. mortality
   mortality within 12 months

   [Time Frame: within 12 months]
5. potentially inadequate medications
   potentially inadequate medications at baseline and for follow-ups at 6 and 12 months

   [Time Frame: baseline and after 6 and 12 months]
6. Adverse Events
   Adverse Events within 12 months

   [Time Frame: within 12 months]
7. Quality of Life in Alzheimer's Disease scale - Nursing Home version (QoL-AD-NH)
   Residents' quality of life at baseline and at 12 months. Scale range is 15 – 60, with higher scores indicating higher QoL.

   [Time Frame: baseline and after 12 months]
8. Health-related Quality of Life (HRQL) of residents measured by EQ-5D-5L
   The EQ-5D-5L consist of two self-rating components: the EQ-5D descriptive system and a visual analogue scale (EQ VAS). The EQ-5D descriptive system measures HRQL on the five dimensions "mobility", "self-care", "usual activities", "pain / discomfort" and "anxiety / depression". On each dimension participants can rate either "no problems" (0), "slight problems" (1), "moderate problems" (2), "severe problems" (3) or "extreme problems" (4), resulting in a score ranging from 0 (best) to 4 (worst). The scores of the single dimensions can be combined to a sum score ranging from 0 (best) to 20 (worst). In addition, so called "utility weights" can be attached to any possible combination of answers on the EQ-5D descriptive system which results in a utility score ranging from 0 (death) to 1 (full HRQL), which is then used to calculate quality-adjusted life years (QALYs). The EQ VAS ranges from 0 (worst imaginable health state) to 100 (best imaginable health state).

   [Time Frame: baseline and after 12 months]
9. medical services
   use of other medical services within 12 months

   [Time Frame: within 12 months]
10. economic evaluation: efficiency
    economic evaluation: efficiency (incremental cost-effectiveness ratio) and cost savings from payer and societal perspective

[Time Frame: after 12 months]

11. Quality of inter-professional collaboration according to the Partnership Self-Assessment Tool (PSAT) score
    Higher score values indicating better collaboration.

    [Time Frame: after 12 months]

12. Attitudes towards inter-professional collaboration according to the Jefferson Scale of Attitudes Toward Physician-Nurse Collaboration (JSAPNC) score
    Higher score values indicating more favourable attitudes.

    [Time Frame: after 12 months]

Eligibility

Minimum Age: 18 Years

Maximum Age:

Sex: All

Gender Based:

Accepts Healthy Volunteers: No

Criteria: Inclusion Criteria Residents:

- at least one GP visit in recent three months or
- two GP visits in recent 6 months or
- admission to the nursing home during the precedent 6 months independently of documented GP contacts
- at least 18 years of age
- written informed consent by the resident or her/his legal guardian

Inclusion Criteria Nursing Homes:

- minimum size of 40 residents
- written consent provided by the nursing home Manager Prior to randomisation

Exclusion Criteria Residents:

- admission for short term care only

Exclusion Criteria Nursing Homes:

- participation in other projects on interprofessional collaboration.

Contacts/Locations

Central Contact Person: Eva Hummers, Prof. Dr. med.
Telephone: +49 551-39-22638
Email: Eva.Hummers-Pradier@med.uni-goettingen.de

Central Contact Backup: Christiane Müller, Dr. med.
Telephone: +49 551-39-22638
Email: christiane.mueller@med.uni-goettingen.de

Study Officials: Eva Hummers, Prof. Dr. med.
Study Principal Investigator
Department of General Practice, University Medical Center Goettingen

Locations: Germany
Department of General Practice, University Medical Center Göttingen

Göttingen, Germany
Contact: Christiane Müller, Dr. med.

Organization and Corporate Development, Georg August University Göttingen
Göttingen, Germany
Contact: Indre Maurer, Prof. Dr.

Institute for Social Medicine and Epidemiology, Nursing Research Group,University of Lübeck
Lübeck, Germany
Contact: Sascha Köpke, Prof. Dr. phil.

Department of General Practice/Primary Care, University Medical Center Hamburg-Eppendorf
Hamburg, Germany
Contact: Martin Scherer, Prof. Dr.

IPDSharing

Plan to Share IPD: No

References

Citations:

Links:

Available IPD/Information:
