# Supplementary material for: Effects of strategies to improve general practitioner-nurse collaboration and communication in regard to hospital admissions of nursing home residents (interprof ACT): study protocol for a cluster randomised controlled trial
Source: Trials. 2020 Nov 5;21:913. doi: 10.1186/s13063-020-04736-x (PMC7643262; doi:10.1186/s13063-020-04736-x)
Supplement: Supplementary file 7 — Additional file 7. Team meetings. [file 13063_2020_4736_MOESM7_ESM.docx]

**Team Meetings in the study interprof ACT**

**Local team meetings:** In each of the three study centres Göttingen, Hamburg and Lübeck the respective teams meet in weekly jours fixes. Here the current state of the study is presented, and next steps are planned on the local levels.

**Multicentre team meetings via web or telephone**: Study team members of all study centres communicate regularly in telephone or online conferences, during the first months weekly, thereafter two-weekly. Topics are current trial state at each centre and next steps. Occurring problems will be handled as well as forming of extra teams for special tasks.

**Multicentre team meetings on site**: All team members meet at one of the trial centres once a year (in the first year 2017 three times) to discuss the main study work packages and milestones: recruitment of nursing homes and residents, as well as GPs and nurses, content of training of interprof ACT agents and kick-off meetings, data collection, data entry and storage, publications strategies, modification of the study process, and implementation and data collection in the context of COVID-19.

**Additional team telephone or online conferences of all centres on special topics are held, on average once a month**: Building consensus on SOPs for data collection, data entry and storage, usage of the database, implementation of the *interprof* ACT programme (trainings and kick-off meeting, supervision of *interprof* ACT agents), and implementation of the intervention in the control group. Moreover, team members attended trainings on the correct process of obtaining the informed consent, conducting standardised interviews and the use of the Secutrial database, and correct performance of Review A (validation of data entry).

**Advisory board meetings:** The advisory board and elected members of the study team meet on average once or twice a year in telephone conferences. The current state of the project is presented, and defined questions are asked, for example: Possible difficulties regarding the implementation of measures, adequate consideration of all involved parties. Additionally general feedback and questions of the advisory board to the study team are part of these meetings.

**Publication committee meetings:** The publication committee - one elected member of each partner (three study centres plus statistics plus health economics)- meets via telephone, once an exposé for a paper or a doctoral thesis is submitted. This committee checks the appropriateness of the content and the authorships and decides whether the writing process is allowed to start.
